# Off-Patent Biological and Biosimilar Medicines in Belgium: A Market Landscape Analysis

**DOI:** 10.3389/fphar.2021.644187

**Published:** 2021-04-19

**Authors:** Yannick Vandenplas, Steven Simoens, Philippe Van Wilder, Arnold G. Vulto, Isabelle Huys

**Affiliations:** ^1^ Department of Pharmaceutical and Pharmacological Sciences, Clinical Pharmacology and Pharmacotherapy, KU Leuven, Leuven, Belgium; ^2^ Ecole de Santé Publique, Université Libre de Bruxelles (ULB), Brussels, Belgium; ^3^ Hospital Pharmacy, Erasmus University Medical Center, Rotterdam, Netherlands

**Keywords:** biological, medicine, competition, sustainability, market access, affordability, Belgium, biosimilar

## Abstract

**Background and objective:** Best-value biological medicines may generate competition in the off-patent biologicals market, resulting in having more resources available to provide patients with access to necessary medicines while maintaining high-quality care. Belgium is a country known to have low biosimilar market shares, suggesting a malfunctioning market for off-patent biologicals. This study aims to gain an in-depth understanding of the Belgian off-patent biologicals market, by looking at the evolution in volumes and costs of the relevant products in the market.

**Methods:** This study included a combination of quantitative and qualitative research methods. The quantitative part of this study consisted of the analysis of market data obtained by the National Institute for Health and Disability Insurance (NIHDI) for all relevant products in the Belgian off-patent biologicals market (i.e. TNF-inhibitors, insulins, granulocyte colony-stimulating factors, epoetins, rituximab, trastuzumab). In addition, for the qualitative part of this study, semi-structured interviews with Belgian stakeholders were conducted between December 2019 and March 2020.

**Results:** Belgian market data and stakeholder perceptions suggest a suboptimal market environment for off-patent biological and biosimilar medicines. Shifts are observed after loss of exclusivities of originator biologicals toward second-generation products or new therapeutic class products, at a higher cost and often limited added value. Moreover, cost reductions for off-patent biologicals after biosimilar market entry are mainly determined by mandatory price reductions applicable to both originator and biosimilar products, and not by lower prices induced by competition. For products used in the retail setting, significant mandatory price reductions for both originator and reference products with low biosimilar volumes were pointed out as the main reasons for the lack of price competition. For products dispensed in hospitals, the hospital financing system is important. First, it does not always encourage the use of lower cost alternatives. Second, competition mainly takes place at the level of confidential discounts in tenders. Most interviewees acknowledged the lack of a competitive environment, which is not supportive of a sustainable Belgian off-patent biologicals market.

**Conclusion:** Market data and stakeholder perceptions indicate that the sustainability of the Belgian market for off-patent biologicals is challenged. A sustainable market ensures access to biological therapies now and in the future.

## Introduction

Over the past decades, biological medicines have led to significant improvement in the treatment of diverse complex, life-threatening, and chronic disorders. Biological medicines or biologicals are large molecules that are produced by living organisms. Because of a complicated development and manufacturing process, biologicals come at a higher cost compared to chemically synthesized products (European Medicines Agency, 2017; Cornes and Bennett, 2018). However, the emerging success of innovative biological therapies has meant a substantial increase in pharmaceutical spending and will continue to put pressure on national healthcare budgets in the coming years.

In Belgium, healthcare spending per capita has grown continuously over the last decades and represented 10.3% of the gross domestic product (GDP) in 2019, which is above the European Union (EU) average of 8.8% (Organisation for Economic Cooperation and Development, 2020). The Belgian national health insurer recognizes the difficult budgetary situation due to the increasing costs of pharmaceuticals year after year. The proposed budget for pharmaceuticals has been exceeded each year over the past five years, with an increase of 7.7% in 2020 and an expected growth of 10.7% in 2021. The total share of pharmaceuticals has increased up to 18% of the overall healthcare budget in 2019 (National Institute for Health and Disability Insurance, 2020a). The aging population, innovative therapies coming to the market in the near future, and new realities such as the recent COVID-19 pandemic will further challenge national healthcare budgets in the near future (Organisation for Economic Cooperation and Development, 2019). As a result, concerns arise on how Belgium will keep its national healthcare system, which is mainly funded by public sources, sustainable in the future.

Biological therapies contribute to an important share of total pharmaceutical spending, with an average of over 30% across Europe in 2020 (IQVIA, 2020). At the Belgian level, biological sales per capita are above the OECD average and higher than its neighboring countries (GaBi online, 2020). The expiration of patents or other original biologicals’ exclusivities offers opportunities for biosimilar medicines to enter the market. A biosimilar medicine is a medicine highly similar to another biological medicine already marketed in the EU (i.e. the reference product) (European Medicines Agency, 2017). For this, biosimilars have proven to have no meaningful clinical differences with respect to their reference product (Cornes and Bennett, 2018). After European market authorization, pricing and reimbursement are regulated on a national level. In Belgium, this is regulated by the federal public service for economics and the National Institute for Health and Disability Insurance (NIHDI). Prices of biosimilars are negotiated on a case-by-case basis, whereby the price of the biosimilar cannot exceed that of the reference product (National Institute for Health and Disability Insurance, 2020c). Consequently, biosimilars introduce competition in the off-patent biologicals market and contribute to a more sustainable healthcare system by reducing costs and providing patients with access to necessary medicines (Vulto, 2019b). To date (February 2021), 34 biosimilar products of 13 distinct molecules are reimbursed by NIHDI and thus marketed in Belgium (Federal Agency for Medicines and Health Products, 2020).

Belgium has a system characterized by significant mandatory price reductions of both biosimilar and reference products after loss of exclusivities, thereby ensuring short-term savings for the national health insurer. The cumulative mandatory price reductions can go up to 38% of the original price (National Institute for Health and Disability Insurance, 2019b; National Institute for Health and Disability Insurance, 2019a; National Institute for Health and Disability Insurance, 2020c). These price reductions may occur earlier when biosimilar medicines enter the market and apply both to reference biological and biosimilar medicines. This measure is called the “biocliff” and entered into force in 2018 (National Institute for Health and Disability Insurance, 2019a). As of July 2020, these price reductions were further enlarged and will apply after 12 years of reimbursement even if no biosimilars have entered the Belgian market (National Institute for Health and Disability Insurance, 2020c).

In addition, different pricing mechanisms apply to biologicals dispensed in public pharmacies and hospitals. On the one hand, in public pharmacies, the net cost for the health insurer equals the list price minus possible co-payments. These prices are transparent and publicly available. On the other hand, biological medicines dispensed in hospitals are subject to tendering procedures, performed by individual hospitals or hospital buying groups. Tenders in the context of biological medicines have the purpose of selecting the most cost-effective supplier(s) for equivalent medicines, as is the case for biosimilar and reference biological products. Therefore, substantial savings are often achieved through confidential discounts when several suppliers exist for equivalent products in tenders (Dranitsaris et al., 2017).

Earlier studies already indicated concerns regarding competition in the Belgian off-patent biological medicines market, characterized by low biosimilar uptake despite implementing a series of *ad hoc* policy measures (Dylst et al., 2014; Moorkens et al., 2020c). This study takes the analysis on the Belgian market a step further than the abovementioned studies, by taking a closer look at the whole Belgian off-patent biologicals market. Because of a potential lack of competition on the market, the benefits of competition may not be fully leveraged in Belgium. The evolution in biosimilar market shares should therefore not be the sole focus. One should also look at the benefits in terms of cost reduction and increase in access to necessary biological therapies, resulting from a competitive market. Because increased competition due to biosimilar market entry may also induce price reductions of the reference product, or even competing products within the same or other therapeutic classes, both biosimilar and original biological products may contribute to these benefits (Vulto, 2019a). As a result, the term *best-value biological* is to be preferred instead of biosimilar or original biological. This emphasizes that the main focus should be on reaching a healthy competition between off-patent and on-patent biological medicines, thereby guaranteeing high-quality care while maintaining an affordable medicines bill (NHS England and NHS Improvement, 2015). This study aims to gain an in-depth understanding of the Belgian off-patent biologicals market. When analyzing the off-patent biologicals market, one should not limit such analysis to off-patent reference and biosimilar products. Other products within the same therapeutic class, as well as competing products of distinct classes are of also importance if one wants to fully understand the functioning of the market.

## Methods

### Quantitative Analysis

In this study, quantitative as well as qualitative results have been obtained. For the quantitative analysis, market data were provided by the Belgian national health insurer (i.e. NIHDI). Market data include sales volumes and total costs. Sales volumes are expressed as Defined Daily Doses (DDD), according to the daily doses defined by the World Health Organization (WHO). Costs are based on the health insurers expenditures plus possible patient co-payments, and do not account for confidential discounts or rebates. These data came from two distinct sources, depending on the setting where the product is dispensed (i.e. public pharmacy or hospital). For products dispensed at the public pharmacy, data were extracted from the Farmanet database, which collects all Belgian data regarding reimbursed medicines in public pharmacies. Market data for products dispensed in hospitals came from the Doc PH database, collecting all data on medication delivery in Belgian hospitals. Market data from 2013 until 2019 were obtained for this analysis. For hospital products, data were only available until June 2019, and were extrapolated until the end of 2019. Data were obtained for off-patent biological medicines and their available biosimilars in Belgium, as well as second-generation biological products, new formulation products of existing biologicals, competing products within the same therapeutic class, or competing products from other therapeutic classes. Molecules included in the market analysis are all off-patent biologicals for which biosimilars are reimbursed in Belgium, and competing molecules within the same therapeutic class (i.e. ATC4 level). Competing molecules from other therapeutic classes were identified during preparatory stakeholder discussions with Belgian stakeholders. For these molecules, it was indicated that significant therapeutic shifts are taking place in Belgium. For products used in the ambulatory care setting, data were also obtained on the therapeutic domain (e.g. gastroenterology, rheumatology, dermatology) where these products were prescribed. Information on the therapeutic domain was derived from data on the prescribing physician’s specialty and was only available for products dispensed in public pharmacies. For each therapeutic class, graphs were designed per product to show evolutions in volume (DDD), total costs (€), and daily costs (€ per DDD) over time. Volume and cost evolutions were analyzed per therapeutic class. For products from other competing product classes, additional figures were designed to show possible therapeutic shifts from off-patent biologicals. For certain products, experts within NIHDI were asked *ad hoc* for additional explanations behind observations in cost evolutions. In this way, specific reasons for price evolutions could be identified.

### Qualitative Analysis

#### Interview Design and Conduct

Semi-structured interviews were conducted with Belgian stakeholders to gain a thorough understanding of the Belgian off-patent biological medicines market. For this, the research team prepared an interview guide based on a literature overview and exploratory discussions with all different stakeholder groups. The interview guide was developed in Dutch, and translated in English and French so all participants could express themselves in their preferred language. Interviewees were given the opportunity to receive the interview guide prior to the interview in order to prepare themselves and provide more in-depth insights. The interview guide consisted of specific parts related to demand-side and supply-side aspects relevant for the off-patent biological medicines market in Belgium (Cfr. Supplementary Material). Two pilot interviews were conducted in advance. Because the interview guide was not modified after this, these interviews were included in the qualitative analysis. At the start of the interview, the researcher presented himself and briefly explained the purpose of the study project. All interviews were conducted by the same researcher (YV) and the same questions were asked in a uniform chronological order to each stakeholder to minimize bias. However, some alterations specific to each stakeholder group were made to ensure only relevant questions were asked. Furthermore, additional questions were asked during the interview to provide participants with the opportunity to clarify certain insights or standpoints. The interviews had a duration of approximately 1 h and were conducted face-to-face or via teleconference, and audio recorded. Each interviewee provided written informed consent prior to the interview for participation to the interview and processing of the data. This study was approved by the Ethics Committee of UZ/KU Leuven (S63406).

#### Participants and Recruitment

A set of predefined inclusion criteria was defined for the selection of interviewees. Eligible participants included physicians, pharmacists (hospital and community pharmacists), nurses, hospital associations, patient organizations, healthcare insurers, and pharmaceutical industry. All stakeholders had to be involved in Belgian policymaking regarding biological medicines by being members of the board of their professional, scientific, patient, or umbrella organization. This ensured sufficient knowledge of each interviewee about the Belgian context and off-patent biological medicines. Physicians working in relevant therapeutic areas for this subject were included (i.e. rheumatology, IBD, dermatology, endocrinology, oncology, hematology, primary care). All healthcare professionals (i.e. physicians, pharmacists, nurses) were required to be closely involved in their professional organization and have experience in daily practice with biological medicines. Hospital representatives had to be part of their umbrella organization and included professionals involved in hospital financing, public procurement, and hospital management. For patient organizations, only Belgian associations regarding relevant therapeutic areas were of interest. Healthcare insurer representatives working at one of the five main Belgian sickness funds and closely involved in policymaking regarding biological medicines were included. Insurance funds are involved in healthcare policy, for example by being part of the reimbursement or insurance committee of NIHDI. Industry participants needed to be involved in market access of off-patent biological or biosimilar medicines or representatives of their Belgian professional organization.

Participants were recruited by means of purposive sampling. This involves identifying and selecting individuals or groups that are especially knowledgeable about the topic of interest, in this case the Belgian market for off-patent biological medicines (Kerr et al., 2010). In addition, participants that met the inclusion criteria could be suggested by other interviewees (snowballing). All participants were invited via e-mail, including an invitation letter with all general information about the study. If they agreed to participate in the study, the information letter and an informed consent form were sent to the participant before the interview. Interviews were conducted until data saturation was reached, meaning no new insights were found (Kerr et al., 2010).

#### Data Analysis

Written transcripts were analyzed following the framework method using Nvivo 12 software (Gale et al., 2013). After transcribing the interviews *ad verbatim* (in the original language), the researcher familiarized himself with the interviews. Subsequently, the first transcripts were coded deductively according to the interview guide themes and prior knowledge through the exploratory discussions and existing literature. Based on additional observations during the coding stage, inductive codes were added to the existing codes. Similar codes were grouped, forming the coding tree. Next, all transcripts were analyzed by categorizing the relevant transcript sections under the related codes. Eventually, all results were summarized in the corresponding framework matrix and interpreted by the researcher.

## Results

### Interviewee Characteristics

In total, 39 interviews were conducted between December 2019 and March 2020 (Table 1). Since multiple interviewees were present at a single interview in most cases, the total number of participating interviewees (*n*) was 55. All stakeholder groups were represented and fitted the inclusion criteria as described under Participants and Recruitment. All healthcare providers (HCPs) were involved in health policy decision making through their professional or scientific association. Participating physicians (*n* = 10) included oncologists, hematologists, rheumatologists, gastroenterologists, dermatologists, and general practitioners. Regarding participating pharmacists, both community (*n* = 2) and hospital pharmacists (*n* = 7) involved in their national professional organization were interviewed. Nurses (*n* = 2) were employed by Belgian hospitals and had experience using biological and biosimilar medicines, more specifically in rheumatology and oncology departments. In addition, they were involved in their professional organization. Patients (*n* = 8) were all affiliated to Belgian patient advocacy groups or patient associations in relevant disease areas, such as hemato-oncology, rheumatology, inflammatory bowel diseases (IBD), and dermatology. Hospital association representatives (*n* = 3) included experts employed by the major hospital umbrella organization in Belgium. Representatives of health insurance funds (*n* = 7) were employed by two of the five major insurance funds in Belgium, covering more than half of the Belgian inhabitants. Industry participants (*n* = 16) were involved in market access and represented both original and biosimilar industry.

**TABLE 1:** Overview of the number of interviews per stakeholder group. The total number of participants is mentioned between brackets.

Stakeholder group	Number of interviews (interviewees)
Physicians	10 (10)
Pharmacists	7 (9)
*Hospital pharmacists*	*5 (7)*
*Community pharmacists*	*2 (2)*
Nurses	2 (2)
Patients	8 (8)
Hospital association	1 (3)
Insurers	2 (7)
Industry	9 (16)
**Total**	**39 (55)**

In order to obtain an overall picture of the Belgian off-patent biologicals market, the results of this study regarding the Belgian off-patent biologicals market will be presented per group of similar molecules or therapeutic class. For each therapeutic class, the results are divided into a section on volume evolution (expressed as DDD) and a section on cost evolution (expressed as cost per DDD). Each section contains the results of the market analysis, supplemented with stakeholder interviews. Therapeutic classes include tumor necrosis factor (TNF) - alpha inhibitors, long-acting insulins, granulocyte colony-stimulating factors (G-CSFs), epoetins, rituximab and trastuzumab. Biosimilars have been marketed in Belgium for each of these classes over the past years. An overview of all products included in the quantitative analysis per therapeutic class, along with their date of market entry in Belgium, is provided as Supplementary Material.

### Tumor Necrosis Factor-Alpha Inhibitors

TNF inhibitors are monoclonal antibodies or fusion proteins designed to bind the chemical messenger TNF-alpha in the human body. TNF-alpha is a cytokine known to cause inflammation and related symptoms in several chronic inflammatory diseases, such as rheumatoid arthritis (RA), ankylosing spondylitis (SpA), psoriatic arthritis (PsA), plaque psoriasis, Crohn’s disease (CD), and ulcerative colitis (UC) (European Medicines Agency, 2014; European Medicines Agency, 2019d; European Medicines Agency, 2020b). TNF inhibitors are reimbursed in Belgium as a second-line treatment for the abovementioned indications after conventional therapy (BCFI, 2020b). In past years, several TNF inhibitors (i.e. infliximab, etanercept, adalimumab) have lost their market exclusivities, allowing biosimilar alternatives to enter the market. Infliximab biosimilars are reimbursed in Belgium since april 2015. For etanercept, the first biosimilar entered the market in September 2016. Since October 2018, biosimilars are available for adalimumab as well. For certolizumab pegol and golimumab, which are still protected by patents, biosimilars are currently under development.

In addition, new competing product classes, such as Janus kinase (JAK) inhibitors and interleukin-17 and -23 (IL-17/23) inhibitors, are influencing this market (Sarzi-Puttini et al., 2019; Kim et al., 2020). Orally administered JAK inhibitors (i.e. baricitinib, tofacitinib, upadacitinib) are approved since 2017 for the treatment of RA, PsA, and UC (European Medicines Agency, 2019c; European Medicines Agency, 2020d). To date, JAK inhibitors are mainly used for the treatment of RA, while most TNF inhibitors have broader indication profiles in rheumatology, dermatology and gastroenterology.

#### Volume Evolution

**FIGURE 1:**
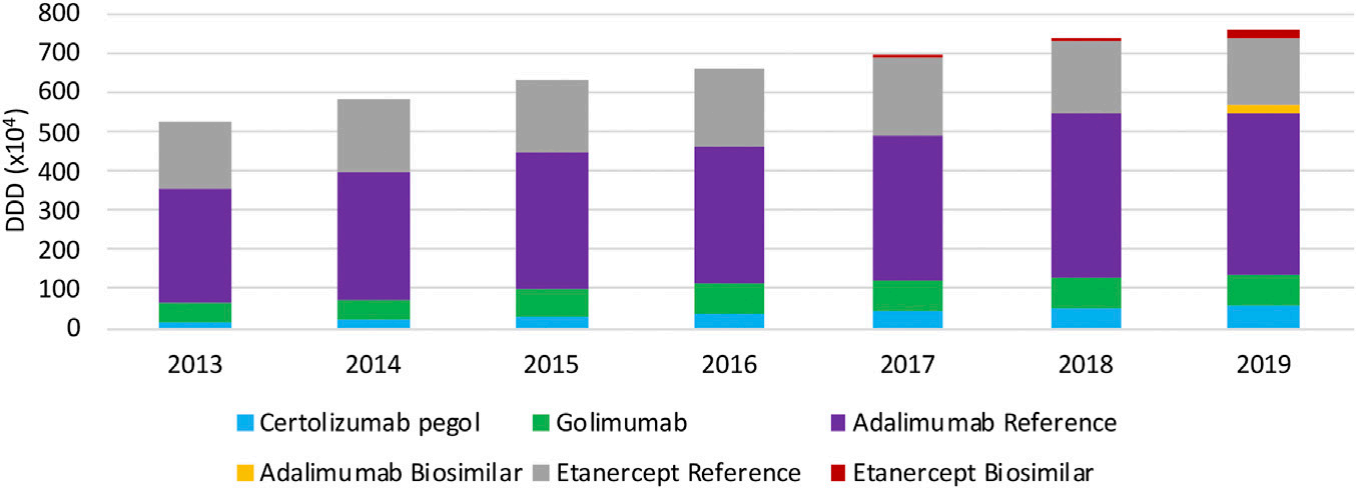
Total volume (DDD) evolution of SC TNF inhibitors between 2013 and 2019.

**FIGURE 2:**
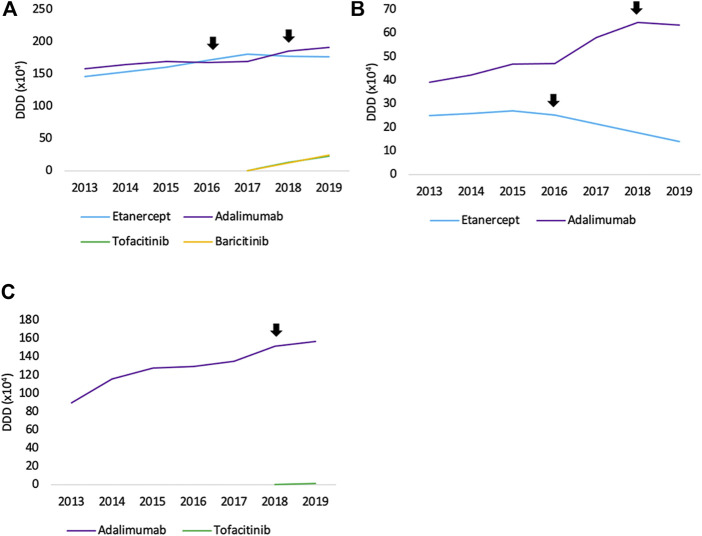
Total volume (DDD) evolution of SC off-patent TNF- and JAK inhibitors per therapeutic area between 2013 and 2019 for **(A)** rheumatology **(B)** dermatology, and **(C)** gastroenterology. Arrows indicate the date of first biosimilar market entry.

**FIGURE 3:**
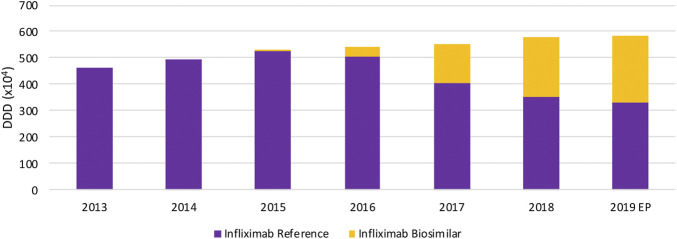
Total volume (DDD) evolution of IV TNF inhibitors between 2013 and 2019. EP: Extrapolated data.

In terms of volume evolution, an increase (+45.4%) in the total volume of SC TNF inhibitors is observed between 2013 and 2019 (Figure 1). This indicates an increase in overall access to TNF inhibitors over the past years. Yet, when looking at each product individually, a few interesting phenomena have been occurring in the past years when looking at both off-patent SC anti-TNFs, etanercept and adalimumab. For etanercept, a decrease in volume right after the first biosimilar market entry was noted. Even though volumes have been increasing the year before. A similar observation applies to adalimumab, although less pronounced, where volumes increased until 2018 and stagnated after biosimilar market entry. However, volume evolutions of etanercept and adalimumab differ per therapeutic domain. Decreasing volumes for etanercept after biosimilar market entry are more pronounced in dermatology and rheumatology. In rheumatology, a simultaneous increase of JAK inhibitor volumes is noteworthy, indicating a shift toward JAK inhibitors. Adalimumab volumes are decreasing as well in dermatology after biosimilar market entry, but not (yet) in the other larger therapeutic domains (Figure 2). Furthermore, volumes of competing anti-TNFs (i.e. golimumab and certolizumab pegol) with less broad indication profiles than adalimumab, have slightly increased over the past years. A steeper increase in the use of certolizumab pegol is being observed in comparison with golimumab. Market shares of certolizumab pegol increased from 3.2% (2013) to 7.4% (2019). Similarly, golimumab has seen its market share growing from 8.4% (2013) to 10.6% (2019). Although increasing market shares of competing on-patent products, this market is still mainly dominated by adalimumab and etanercept (82.0% of the total market in 2019). Biosimilar market shares of etanercept, with respect to the reference product, increased to 12.5% in 2019. Adalimumab biosimilars have limited market shares of only 5.2% in 2019. When considering the total SC anti-TNF market, these market shares are negligible, especially for adalimumab. Regarding the only IV administered product within this class, infliximab, volumes have been increasing continuously between 2013 and 2019 (Figure 3). Most recent market data suggest a more balanced market with a 43.7% infliximab biosimilar market share in 2019.

Interviewed physicians indicated that a minority of new patients is initiated with SC TNF inhibitors nowadays, particularly for dermatology and rheumatology indications. Dermatologists indicated their preference for IL-17/23 inhibitors for newly treated patients with psoriasis. Unfortunately, no reimbursement data were available for IL-17/23 inhibitors at the time of the analysis. Rheumatologists mentioned a trend toward preferential prescribing of JAK inhibitors since their market entry in 2017. Both statements are supported by the market data. It may be that adalimumab volumes will follow a similar trend as etanercept during 2020 since a delay of one year was observed for etanercept as well. Gastroenterologists mentioned the smaller importance of competing products such as IL- and JAK inhibitors in their therapeutic area. They have a less important place in the treatment of IBD, which is reflected in the observations based on the reimbursement data.

Belgian stakeholders acknowledged that biosimilars remain needed in the future to generate competition in the market. The lack of incentives to compensate for additional efforts to transition to a biosimilar, the minor price differences between biosimilars and reference products dispensed in the retail setting, and the lack of transparency about the generated savings due to increased competition were pointed out as important disincentives for biosimilar usage. The reasons for the prescribing behavior with shifts to newer and often more expensive alternatives (e.g. JAK inhibitors) remain unclear at the moment. The possible increased user-friendliness of the oral administration of JAK inhibitors might be one of the factors contributing to the observed prescription behavior.

#### Cost Evolution

**FIGURE 4:**
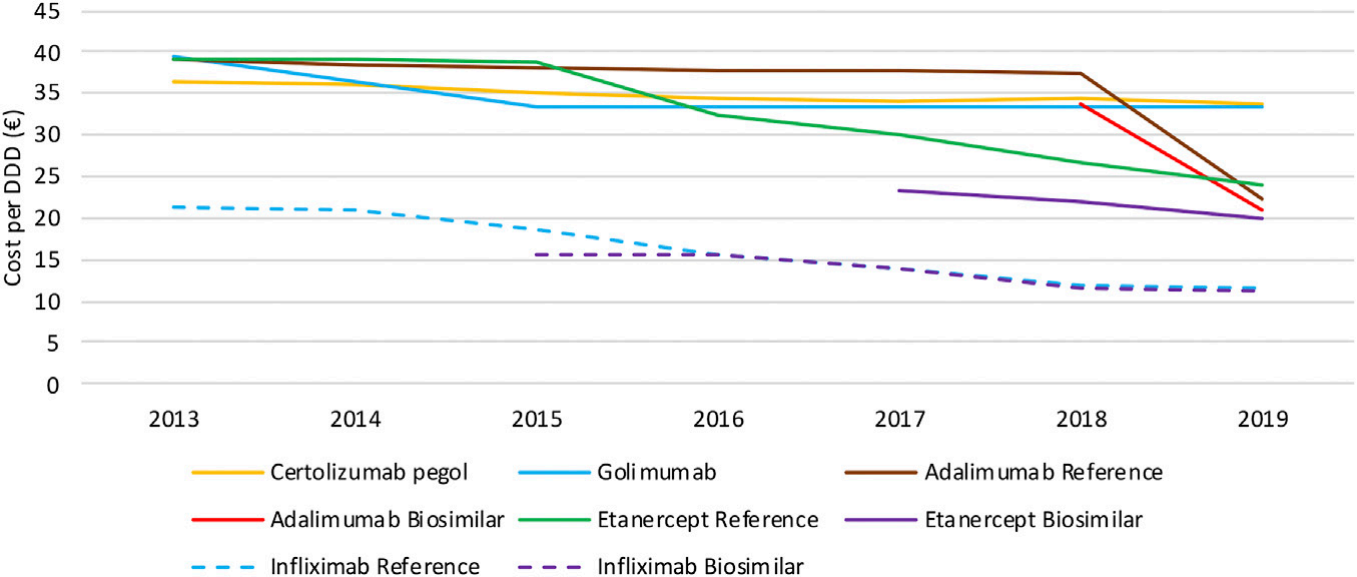
Daily cost (€ per DDD) evolution of TNF inhibitors between 2013 and 2019.

When looking at the evolution in daily costs of SC anti-TNF in the observed period, substantial reductions are observed after biosimilar market entry for all off-patent biologicals (Figure 4). For etanercept, the stepwise application of a series of mandatory price reductions for both the reference product and the biosimilar is responsible for the daily cost reduction of 39.6% since biosimilar market entry. Daily costs of adalimumab dropped substantially (−41.2%) after its loss of exclusivities in 2018 due to the simultaneous application of several mandatory price reductions. In general, daily costs of SC TNF inhibitors are almost exclusively decreasing because of mandatory price reductions. Additional cost savings because of price competition do not occur for this market in Belgium over the observed period of time. Moreover, all price reductions have been applied to both biosimilar and reference products, resulting in only minor price differences between reference and biosimilar products in Belgium. Daily costs of competing products golimumab and certolizumab pegol approximately remained constant over time. In addition, there are differences in treatment costs in Belgium between TNF- and JAK inhibitors. On-patent TNF inhibitors (i.e. certolizumab pegol and golimumab) and JAK inhibitors cost on average 48.3% and 40.6% more than off-patent SC anti-TNFs. This approximately equals the price difference for etanercept and adalimumab before and after biosimilar market entry, meaning shifts toward these new products largely offset savings generated after biosimilar market entry. Daily costs of all products included for this study in 2019 are included in the Supplementary Material.

Intravenously administered infliximab is the only TNF inhibitor exclusively dispensed in hospitals. The daily cost calculated for infliximab is based on the reimbursement data, and thus reflects the evolution of the reimbursement base. Several mandatory price reductions after biosimilar market entry were responsible for the observed daily cost reduction of 47.3% between 2013 and 2019 (Figure 4). The only spontaneous price decrease (−3.3%) was observed for infliximab originator Remicade in 2015 to align their price with the lower priced biosimilars. All other price reductions were part of mandatory price reductions or cost saving measures.

Interviewed stakeholders pointed at the importance of a balanced market in order to generate price competition among SC TNF inhibitors. The small biosimilar market shares do not offer companies the opportunity to lower their prices spontaneously. A particular volume of biosimilar products is required to induce lower prices and establish a competitive environment. Furthermore, because of the already significant mandatory price reductions for both biosimilar and reference products, the room for further price reductions is limited at such low volumes. The current Belgian system with mandatory price reductions was seen as a way to secure short-term savings in the off-patent biologicals market. However, without volume guarantees for biosimilars, this system is not sustainable in the future.

The situation for intravenously administered infliximab was considered different from SC TNF inhibitors, because of its exclusive use in the hospital setting. Interviewees mentioned two main reasons for the lack of price competition on the reimbursement base or list price for infliximab. In fact, these reasons are not specific to infliximab and apply to all hospital biological products. First, price competition occurs on net prices through confidential discounts in tender procedures and are therefore not reflected in the obtained reimbursement data. Second, the generated savings due to the difference between the reimbursement by the national health insurer and net price paid by hospitals, contribute to the hospital financing. That is why hospitals are interested in selecting the product with the largest difference between the net and reimbursed prices. This results in a situation where lowering list prices is discouraged, as this creates a disadvantageous position in tenders. As acknowledged by the interviewees, the interference with the hospital financing system is therefore imperative in this regard. The optimization of the Belgian hospital financing is not a topic that will be discussed here. Nonetheless, it is known to strongly influence the off-patent biologicals market in Belgium and certainly deserves the attention.

### Long-Acting Insulins

Insulins are essential for the treatment of a growing population of type I and type II diabetes mellitus (DM). Basal insulins refer to longer-acting insulin intended to cover the body’s basal metabolic insulin requirement, including both intermediate- and long-acting insulins (Davies et al., 2018). They are the preferred initial insulin for patients with type 2 DM. During the past decade, long-acting insulin analogs have been introduced into the market in addition to existing intermediate-acting human insulin (i.e. insulin NPH). Long-acting insulin analogs include insulin glargine, insulin detemir, and insulin degludec and were developed with the intention to achieve more stable glycemic control (Goldman et al., 2017). Insulin glargine, the first product in this class, has two different dosage forms on the market administered once daily. The traditional 100 U/ml formulation (Lantus) and the more concentrated 300 U/ml (Toujeo) that is reimbursed in Belgium since October 2016. A biosimilar of insulin glargine 100 U/ml (Abasaglar) is available in Belgium since 2016 as well. A second molecule within this class is insulin detemir (Levemir) and was marketed in Belgium as an alternative to insulin glargine in 2005. The third and most recent product among long-acting insulin analogs is insulin degludec (Tresiba) and was marketed in Belgium in april 2019.

#### Volume Evolution

**FIGURE 5:**
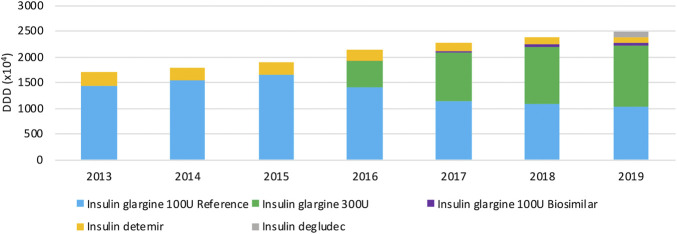
Total volume (DDD) evolution of long-acting insulins between 2013 and 2019.

Figure 5 provides an overview of the volume evolution of different products within this therapeutic class, dispensed in public pharmacies between 2013 and 2019. Overall, volumes of long-acting insulin analogs have been increasing continuously over the past years. Insulin glargine is prescribed most within the class of long-acting insulins, taking up the majority of the market. A preference for the insulin glargine reference product, and the more concentrated formulation after its market entry in 2016, is clearly observed. A rapid increase in the insulin glargine 300 U/ml market share is observed in recent years, to 47.0% in 2019. The market share of insulin glargine 300 U/ml might be slightly overestimated though, because the concentrated formulation is less efficient and may require higher doses (10–14%) compared to insulin glargine 100 U/ml, while the same DDD is being used. Insulin detemir is used to a lesser extent (4.7% of the total market in 2019), with decreasing volumes over the studied period. The insulin glargine biosimilar has limited use and a market share of only 2.2% of the total market, and 5.0% of all insulin glargine 100 U/ml in 2019. As a result, originator products of insulin glargine make up 89.1% of the total market in 2019. Although insulin degludec was marketed only in April 2019, already a market share of 4.0% is achieved.

The most important molecule within this class, insulin glargine, has three different products. However, the market is largely dominated by originator products. Interviewees confirmed that this situation is not desirable and that biosimilars remain needed to induce competition within this therapeutic class. The minor price difference was pointed out by interviewees as an important disincentive to prescribe biosimilars for economic reasons, both for new patients and patients already treated with long-acting insulins. It was acknowledged that a certain volume for biosimilars will be required to reduce costs in the future, especially with the prospect of other insulins soon facing biosimilar competition (e.g. insulin aspart) and the increasing financial burden of diabetes treatment. Loyalty to originator products may also play an important role and could explain the hesitancy to prescribe biosimilar insulins. Similar to TNF inhibitors, another reason not to switch to a biosimilar for economic reasons is the additional time investment, for example because of differing injection devices.

#### Cost Evolution

**FIGURE 6:**
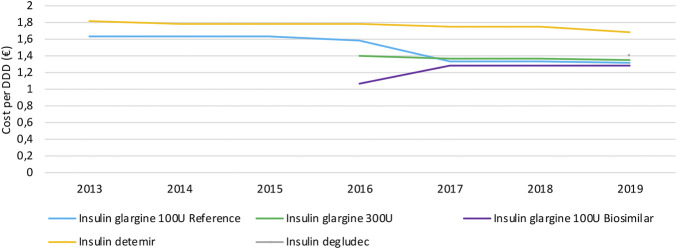
Daily cost (€ per DDD) evolution of long-acting insulins between 2013 and 2019.

Similar to the observations for TNF inhibitors, daily costs of long-acting insulin products mainly decrease because of mandatory price reductions to both reference and biosimilar products after biosimilar market entry (Figure 6). Daily costs of insulin glargine 100 U/ml decreased by 18.9% due to biosimilar market entry in 2016. No decrease in daily costs is observed for insulin glargine 300 U/ml, neither for second-generation insulin detemir. Insulin glargine products approximately have the same cost, with a slightly lower cost for insulin glargine 100 U/ml. However, insulin glargine 300 U/ml is expected to cost more on a daily basis than calculated here, because the higher required dosage of 10–14% compared to insulin glargine 100 U/ml is not accounted for in this analysis. Hence, the cost per DDD for the more concentrated formulation might be underestimated and actual cost differences could therefore be larger than calculated based on these data. Second-in-class product insulin detemir is the costliest treatment option in this therapeutic class with an average daily cost of 28.1% more compared to insulin glargine. Insulin degludec comes at approximately the same cost as insulin glargine, with a 6.3% higher average daily cost.

### Granulocyte Colony-Stimulating Factors

Granulocyte Colony-Stimulating Factors (G-CSFs) are growth factors used for the prophylaxis of chemotherapy-induced neutropenia. Short-acting G-CSFs can also be used to reduce neutropenia in patients undergoing a bone marrow transplant (European Medicines Agency, 2019b; European Medicines Agency, 2019a). There are two main subtypes of G-CSF products, the short-acting filgrastim and long-acting pegfilgrastim or lipegfilgrastim. Filgrastim has been available for almost 2 decades and multiple biosimilars are available in Belgium since January 2010. Second-generation product pegfilgrastim recently lost its exclusivities, enabling biosimilars to enter the Belgian market as of April 2019. However, pegfilgrastim biosimilars were not yet included in this analysis since data were only available until June 2019 for products dispensed in hospitals. In addition, the second-in-class long-acting G-CSF lipegfilgrastim entered the market in 2014. G-CSFs are mainly subject to tender procedures by hospitals or hospital buying groups.

#### Volume Evolution

**FIGURE 7:**
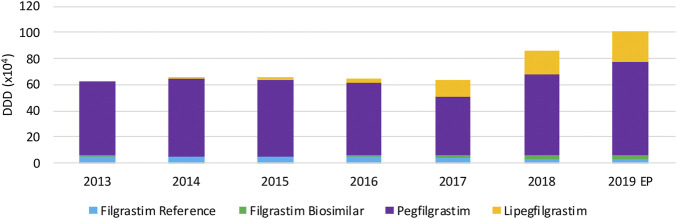
Total volume (DDD) evolution of G-CSFs between 2013 and 2019. EP: Extrapolated data.

Total usage of G-CSFs remains constant over the period between 2013 and 2017 (Figure 7), whereas a remarkable increase in total volume is observed in 2018 and 2019, particularly because of the increasing use of lipegfilgrastim and pegfilgrastim. It is clear that this market is dominated by long-acting G-CSFs, covering 94.3% of the market. Lipegfilgrastim market shares increased especially during the last years to 23.6% of the total market. A steep increase in pegfilgrastim volumes in 2018 and 2019 is observed after an unexpected decline in 2017. Despite a shift toward lipegfilgrastim, pegfilgrastim retained its dominant position with 70.7% of the total market in 2019. Filgrastim volumes remained constant, but its share decreased to 5.7% of the total market in 2019. Regarding biosimilar market shares over the studied period, the market share of filgrastim biosimilars has sharply increased from 4.9% in 2016 to 46.6% in 2019, with respect to the reference product. However, because short-acting filgrastim is only a minor part of the G-CSF market, biosimilar market shares remain low with respect to the total market (2.7%). Data for pegfilgrastim biosimilars, which entered the market in april 2019, were not available since this analysis included data until June 2019.

#### Cost Evolution

**FIGURE 8:**
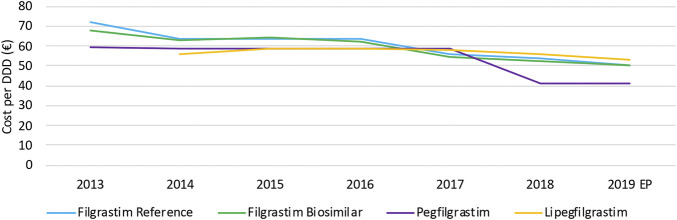
Daily cost (€ per DDD) evolution of G-CSFs between 2013 and 2019. EP: Extrapolated data.

The decrease in daily costs of filgrastim (−12.1%) due to the lowering of the reimbursement in 2017 is observed in Figure 8. Long-acting G-CSFs show no price decrease until 2017, after which daily costs decreased especially for pegfilgrastim. The observed decrease in daily costs for pegfilgrastim in 2018 (−29.8%) resulted from of the mandatory price reduction after 12 years of reimbursement. In addition, a further lowering (−16.0%) was the result of a twofold spontaneous price reduction of the reference product in 2018. However, an additional decrease in daily costs for pegfilgrastim is expected in 2019 due to mandatory price reductions because pegfilgrastim biosimilars entered the market. Unfortunately, this analysis included data until June 2019 so this could not yet be observed.

As already discussed earlier for infliximab, interviewees mentioned that price competition does not occur on the level of list prices for hospital products, and that price reductions are mainly the result of cost-containment policies. However, the situation for G-CSFs is more complex compared to infliximab. Generally, the reimbursed prices are aligned for the reference product and the biosimilar to achieve a fair tender process. This is not the case for competing products with different ATC-codes, such as pegfilgrastim and lipegfilgrastim. Most Belgian hospitals tender for long-acting G-CSFs in the same parcel, acknowledging the equivalence of both products. However, list prices of both products with different ATC-codes differed, which is the main reason why many hospitals preferred lipegfilgrastim instead of pegfilgrastim in tenders. The higher reimbursed price for lipegfilgrastim compared with pegfilgrastim provided lipegfilgrastim with a competitive advantage in tenders. Meanwhile, the reimbursement of pegfilgrastim and lipegfilgrastim has been aligned in May 2020 to solve this issue. Yet, the price of pegfilgrastim products was subsequently lowered further in July 2020 because of a mandatory price reduction. This generated a new price difference between both second-generation G-CSFs. This gave lipegfilgrastim again a competitive advantage in tenders with respect to pegfilgrastim. As mentioned by the interviewees, these elements have disrupted the Belgian market of long-acting G-CSFs in recent years.

### Epoetin

Epoetin, the biosynthesized recombinant form of human erythropoietin, stimulates the production of red blood cells and is therefore used for the treatment of anemia. In normal circumstances, erythropoietin is produced by the kidneys. However, in patients undergoing chemotherapy or suffering from chronic renal disorders this production might be suppressed, resulting in anemia (European Medicines Agency, 2018a; European Medicines Agency, 2018b; Santos et al., 2019). Several epoetin products are marketed in Belgium. Short-acting epoetins alpha (Eprex, Binocrit), beta (NeoRecormon), and zeta (Retacrit) are available on the Belgian market for more than a decade. Binocrit and Retacrit are developed as biosimilar versions of the reference product Eprex, generating competition after its loss of exclusivities in 2008. Meanwhile, second-generation products have been developed with prolonged action. In 2002, darbepoetin alpha (Aranesp) was marketed in Belgium as the first epoetin with extended duration (Deicher and Hörl, 2012; European Medicines Agency, 2020a). Remarkably, biosimilar versions of darbepoetin alpha are only marketed in Japan and not in Europe, despite its patent expiry already in 2016 (GaBi online, 2019c). Later, in 2008, a methoxy polyethylene glycol (MPG) conjugate of epoetin beta has been developed under the brand name Mircera to achieve a lower administration frequency compared to existing short-acting alternatives (Curran and McCormack, 2012). All epoetins in Belgium are exclusively used in the hospital setting and compete therefore through public procurement procedures.

#### Volume Evolution

**FIGURE 9:**
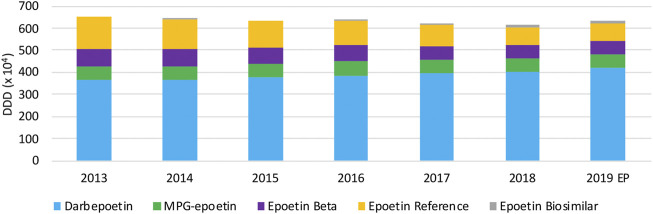
Total volume (DDD) evolution of epoetins between 2013 and 2019. EP: Extrapolated data.

Over the period 2013 until 2019, as can be observed in Figure 9, total volumes slightly decreased (−5.6%) until 2018. In 2019, a modest increase (2.7%) in total epoetin volumes was noted. Long-acting epoetins dominate the overall epoetin market with increasing market shares from 64.8% (2013) up to 76.1% (2019), in particular because of increasing market shares of darbepoetin. Among short-acting epoetins, the decreasing trend in volumes of epoetin alpha is noteworthy. Biosimilars for the reference product of epoetin alpha have negligible market shares, despite their market entry already in 2008. Volumes of other competing products MPG-epoetin and epoetin beta remained constant, with market shares close to 10% of the total market for each product in 2019.

The observed decrease in total epoetin volumes between 2013 and 2019 was reported to be because of new dosing guidelines for all epoetins. This decrease was already observed the years before 2013 in Belgium, similar to the findings of a regional analysis in Italy over the same period of time (Ingrasciotta et al., 2015; National Institute for Health and Disability Insurance, 2018). It became clear from interviewed stakeholders that most Belgian hospitals tender separately for long and short-acting epoetins, in order to achieve the most economically interesting bid. This generates competition among short-acting epoetins, with biosimilars and originator products, as well as among two long-acting originator products. Even though it was confirmed by most interviewees that there is a clear need for biosimilar medicines to generate competition among short-acting epoetins and the resulting lower net prices in tenders, their use remains limited in Belgian hospitals. Some hospitals make specific parcels per indication, since reimbursed or approved indications differ between products. A preference for long-acting darbepoetin alpha was indeed mentioned by clinicians and pharmacists, as observed in the market data. The fact that fewer administrations are needed, was seen as a major benefit in terms of efficiency and patient quality of care.

#### Cost Evolution

**FIGURE 10:**
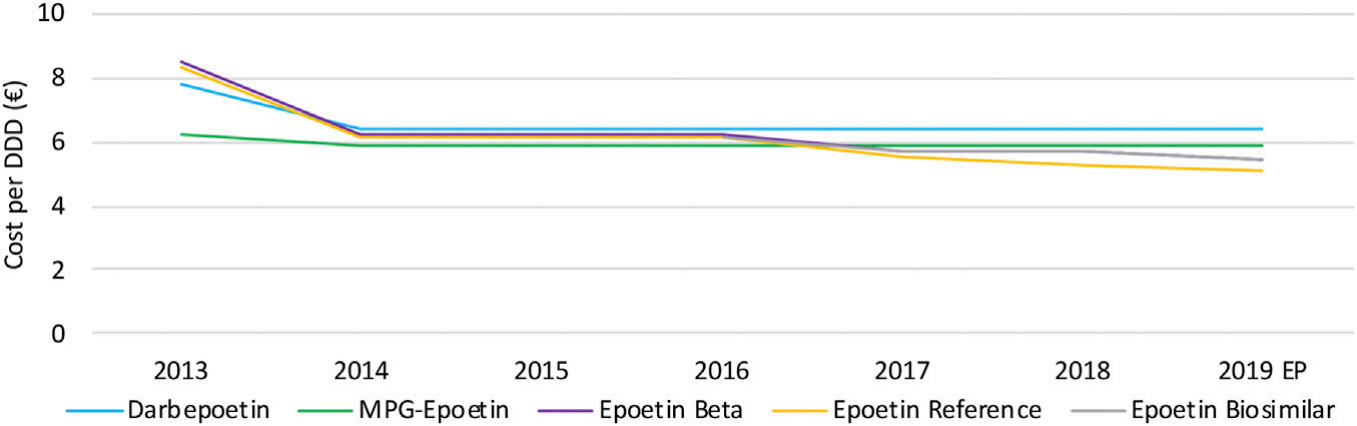
Daily cost (€ per DDD) evolution of epoetins between 2013 and 2019. EP: Extrapolated data.

Daily costs of epoetins have especially decreased in 2014 and 2017, because of two distinct cost-containment measures (Figure 10). The decrease in 2014 is due to the lowering of the flat-rate reimbursement for all epoetins. This led to daily cost reductions of short-acting epoetins (−16.0%), darbepoetin alpha (−17.2%), and MPG-epoetin (−5.6%). A second daily cost decline is observed in 2017 due to the lowering of the reimbursement with 10% for short-acting epoetins, leading to a minor reduction for short-acting epoetins (−11.3%) in 2017. Since flat-rate reimbursements were aligned for all short-acting epoetins, daily costs are expected to be approximately the same. Long-acting products darbepoetin alpha and MPG-epoetin are on average 22.0% and 11.1% more expensive than short-acting epoetins, based on the most recent market data. Further non-imposed price reductions are not observed for this therapeutic class.

During the past decade, several changes were made regarding reimbursement modalities for epoetins in Belgium. Examples are the introduction of the lump-sum reimbursement and the flat-rate reimbursement at the level of the cheapest product for all epoetins. This has resulted in a halving of the overall costs for epoetins between 2011 and 2016 (National Institute for Health and Disability Insurance, 2018). Most interviewees saw the current system of lump-sum reimbursement for this therapeutic class as a way to stimulate the purchasing of lower cost medicines. However, the dominant position of certain products because of their unique indication profile would lead to less advantageous prices. The persistent reduction of the lump-sum reimbursement over time was seen as a major issue in this regard, hindering a correct prediction of the hospital revenues for pharmaceutical products. The influence of hospital financing in Belgium is again important in this case. Nonetheless, it was generally accepted that a lump-sum reimbursement is advantageous to stimulate price awareness when making tender decisions, if these are set up fairly and not continuously tightened as is the case now. As a result, questions were raised whether this market would remain sustainable if cost-containment policies are introduced repeatedly.

### Rituximab

Rituximab is a monoclonal antibody (mAb) that recognizes and binds the CD20 protein on the surface of B-lymphocytes. This causes depletion of B-lymphocytes, which is beneficial in case of B-cell malignancies (i.e. hematological cancers) or in diseases where B-cells cause inflammation (i.e. rheumatoid arthritis). It is therefore used for the treatment of several types of hematological cancers (i.e. follicular lymphoma, non- Hodgkin’s lymphoma, chronic lymphocytic leukemia (CLL)) and severe autoimmune diseases (i.e. RA, granulomatosis with polyangiitis and microscopic polyangiitis) (Santos et al., 2019; European Medicines Agency, 2020c). Rituximab was initially administered only intravenously, but a SC injection is also available in Belgium since December 2014. The SC formulation is approved for the same indications as the IV formulation, except for RA. The first rituximab biosimilar (Truxima) is reimbursed since November 2017. Rituximab is exclusively dispensed in hospitals and is therefore subject to tender procedures.

#### Volume Evolution

**FIGURE 11:**
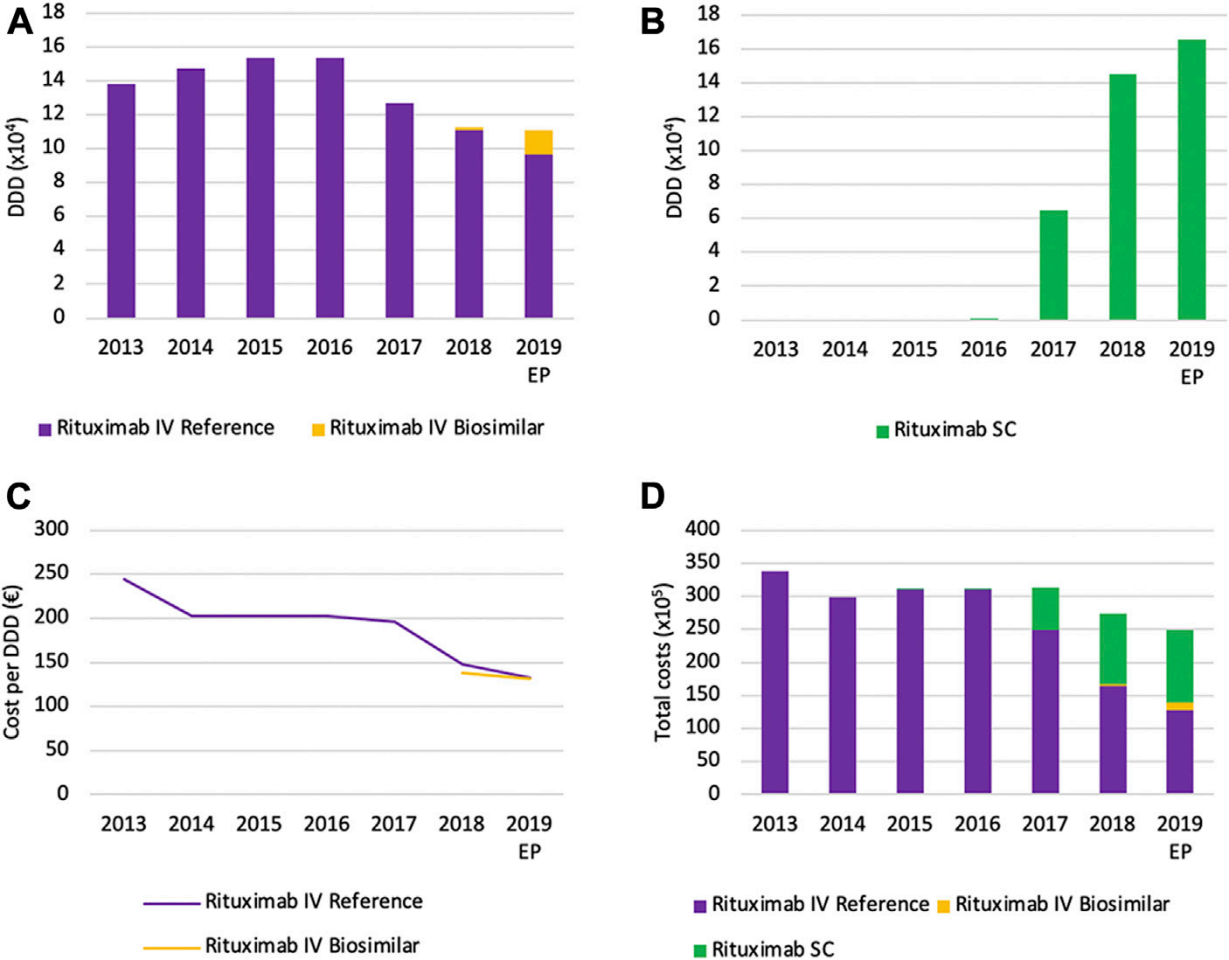
Market data evolution of rituximab between 2013 and 2019. Total volume (DDD) evolution of IV rituximab **(A)**; Total volume (DDD) evolution of SC rituximab **(B)**; Daily cost (€ per DDD) of IV rituximab **(C)**; Total cost (€) evolution of rituximab **(D)**. EP: Extrapolated data.

Volumes for intravenously administered rituximab increased continuously between 2013 and 2016, and suddenly decreased thereafter with 27.7% between 2016 and 2019 (Figure 11A). The main reason for this observation is the increased use of the SC formulation of rituximab instead of the IV formulation, as can be observed through the increasing volume of the SC formulation (Figure 11B). However, because the posology is different between SC and IV formulations, caution is advised with the interpretation of DDDs. The DDD for rituximab is calculated based on the IV posology, a comparison with the SC version based on the same DDD would therefore not be appropriate. Unfortunately, there were no data available about the total number of patients for each product. This would have allowed making comparisons between IV and SC usage. Nonetheless, volumes in terms of DDDs for the SC version are still informative to see trends in volume evolution. Because of the delayed market entry of biosimilar products for rituximab in 2017, biosimilar market shares are negligible in 2017 and 2018. However, biosimilar volumes seem to increase rapidly to 12.7% of the IV market in 2019.

#### Cost Evolution

Daily costs for rituximab IV have decreased (−45.9%) between 2013 and 2019, particularly in 2014 and 2018 (Figure 11C). The main reason for this decrease is the introduction of cost-containment measures in 2014, 2018, and 2019. The application of the measure “old medicines” resulted in a corresponding price reduction of 17.1% in 2014. This cost reduction was accompanied by the broadening of the reimbursement conditions for rituximab, providing access to this treatment for more patients (National Institute for Health and Disability Insurance, 2018). The second cost reduction (−24.9%) in 2017 resulted from the application of mandatory price reductions due to biosimilar market entry. In addition, daily costs decreased further (−10.3%) in 2019 due to a subsequent decrease of the reimbursement as a new cost-containment measure. Illustrative for the increasing use of subcutaneously administered rituximab is the fact that the SC formulation accounts for 42.7% of the total expenses for rituximab in 2019 (Figure 11D). This could serve as a proxy for the market share of the SC formulation, since costs per treatment cycle for the health insurer have been aligned for SC and IV formulations (National Institute for Health and Disability Insurance, 2014b).

### Trastuzumab

Trastuzumab is a mAb for the treatment of several types of cancers that overexpress the human epidermal growth factor receptor 2 (HER-2). Overexpression of HER-2 causes uncontrolled cellular proliferation and occurs in about a quarter of all breast cancers and a fifth of gastric cancers. Trastuzumab binds to HER-2, thereby stimulating the immune system and inhibiting tumor growth. Trastuzumab is used in combination with other chemotherapeutic products, in both metastatic and early stage cancers (European Medicines Agency, 2013; Barbier et al., 2019). Similar to rituximab, there are two different administration routes for trastuzumab currently available. After the IV version (Herceptin) was marketed in 2002, a SC version is reimbursed in Belgium since July 2014. Biosimilars of trastuzumab are marketed in Belgium since August 2018.

#### Volume Evolution

**FIGURE 12:**
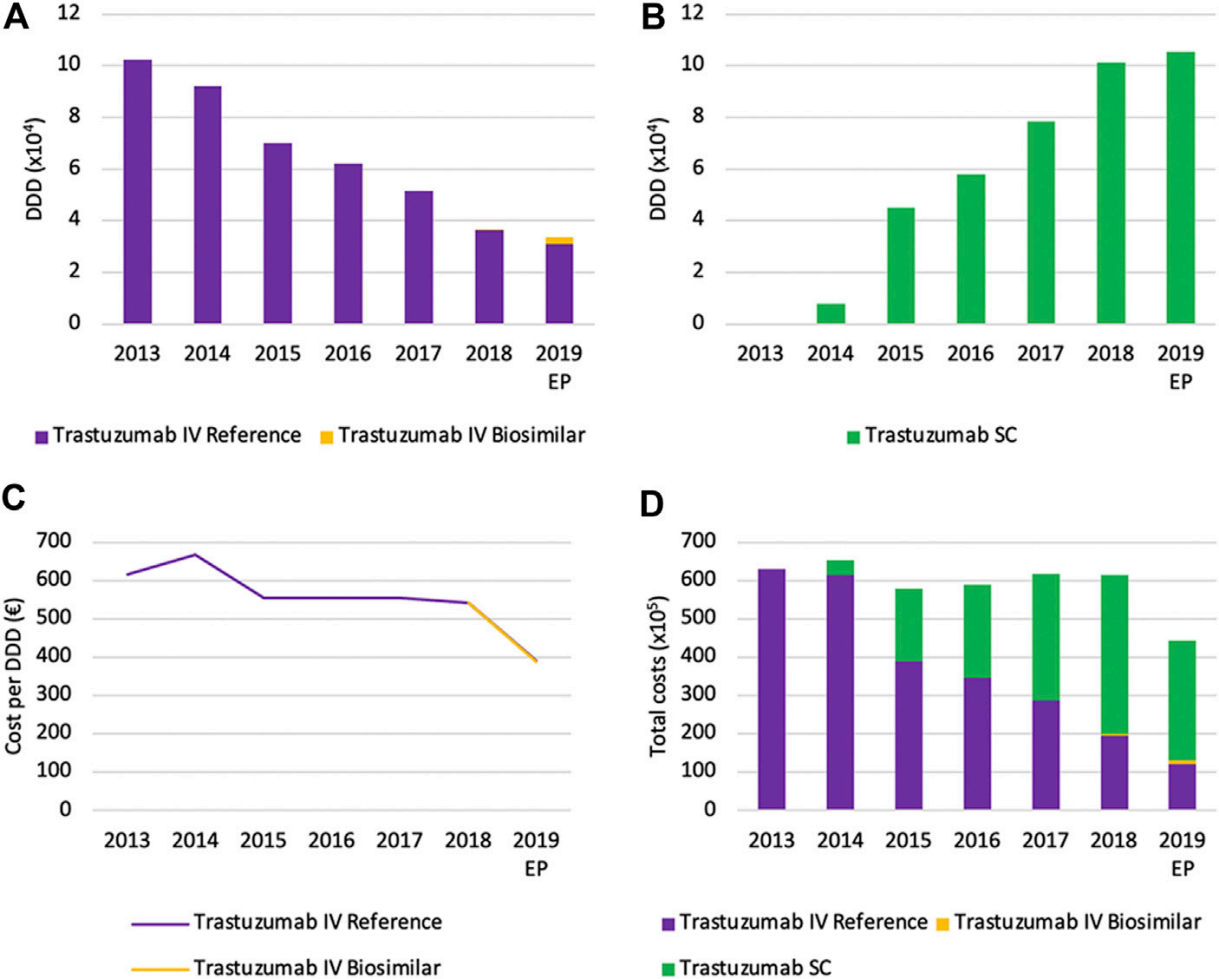
Market data evolution of trastuzumab between 2013 and 2019. Total volume (DDD) evolution of IV trastuzumab **(A)** Total volume (DDD) evolution of SC trastuzumab (**B**) Daily cost (€ per DDD) of IV trastuzumab **(C)** Total cost (€) evolution of trastuzumab **(D)**. EP: Extrapolated data.

Total volumes of IV trastuzumab are decreasing rapidly right after the market entry of the SC version, with a decrease from 2013 until 2019 of 67.1% (Figure 12A). Similar to rituximab, the mean reason for this decrease is the shift toward the SC version of trastuzumab, as can be observed by the prompt increase of SC volumes (Figure 12B). Again, volumes (DDD) of IV and SC formulations should not be compared directly because of a different posology. The shift toward subcutaneously administered trastuzumab seems to be more explicit compared to rituximab. This could partly be explained by the identical indication profile of both SC and IV formulations, which is not the case for rituximab. Furthermore, treatment with trastuzumab does not require to be initiated with an intravenous administration, as is the case for rituximab (European Medicines Agency, 2013; European Medicines Agency, 2020c). Biosimilar market shares of trastuzumab remain low with a market share of 8.5% of the total IV market in 2019.

In general, the market situation for rituximab and trastuzumab were assumed to be similar by the interviewees. During the interviews, it was mentioned several times that Belgian rheumatologists, hematologists and oncologists do not consider any differences in clinical outcomes between SC and IV formulations, nor between reference and biosimilar products. Other factors play a role in the rapid uptake of SC formulations and low biosimilar usage in Belgium. Increased efficiency in terms of patient waiting times and administration duration were considered to be the main reasons for hospitals to prefer the SC administration. However, interviewees pointed out the possible difference between larger and smaller hospitals, with differing daycare capacity. For smaller hospitals, the increased efficiency of the SC formulation might be more meaningful compared to larger academic hospitals. Because more patients can be treated daily, more revenues are expected for the hospital. For this reason, the influence of the hospital financing system was again indicated as being of importance. Yet, this only explains the shift toward SC formulations, and not the dominance of reference products among intravenously administered rituximab and trastuzumab. The observation that biosimilar market shares remain low among IV products may be due to the preference of having a single supplier of the same product for logistic reasons in hospitals. Another possible reason could be the tied selling of SC and IV products in tenders, as suggested by some interviewees, where additional discounts were provided for the SC formulation. Thirdly, patent disputes for rituximab and a general hesitancy to tender early by Belgian hospitals were indicated as explanations for the slow and limited biosimilar uptake of rituximab and trastuzumab.

#### Cost Evolution

Daily costs of IV trastuzumab remained approximately constant between 2013 and 2018, after an initial decrease (−16.7%) in 2015 because of the mandatory price reduction of after 12 years of reimbursement (Figure 12C). A larger decrease in daily costs in 2019 (−27.9%) is the result of mandatory price reductions following the availability of biosimilars. No further spontaneous daily cost reductions were observed in the studied period of time. Total costs for trastuzumab are mainly determined by the increased use of the SC formulation in recent years (Figure 12D). In 2019, costs of SC trastuzumab were over two-thirds of the total costs (70.3%). Analogous to rituximab, the cost per treatment was equated for SC and IV trastuzumab during the reimbursement procedure (National Institute for Health and Disability Insurance, 2014a). As a result, the cost-share of the SC formulation underlines its dominant position on the Belgian market.

For both rituximab and trastuzumab, interviewees mentioned again that competition does only occur on net prices in tenders. Hospital pharmacists expressed other possible benefits of trastuzumab biosimilars in addition to reduced costs. Several biosimilars offer the additional benefit of providing a larger dosage form (420 mg, in addition to 150 mg), allowing hospital pharmacists to better tailor the available pack sizes to the individual patient’s needs. With the optimal use of the two package sizes, resources are used more efficiently with associated savings for the Belgian healthcare system. Similar small improvements may help biosimilars to generate an additional competitive advantage with respect to their competitors.

## Discussion

This article is the first to provide an overview of the market dynamics within the Belgian off-patent market for biological medicines, by looking at reference, biosimilar, second-generation, and other competing products. Insights of different stakeholders accompany market data in order to capture a complete image on how the Belgian market behaves. Off-patent biologicals and their competing products were analyzed per therapeutic class, thereby considering varying dynamics between these classes. Volume and cost evolutions were investigated for all relevant products within each class. This study did not aim to formulate specific policy recommendations to overcome the identified barriers, as already addressed in a recent publication about the Belgian biosimilar landscape (Moorkens et al., 2020c).

### Price Competition

#### Retail Setting

In Belgium, most off-patent biologicals and biosimilars are used in the hospital setting and are therefore subject to tendering procedures. Two major classes of biologicals dispensed in public pharmacies are subcutaneously administered TNF inhibitors and insulins. In 2019, Belgian total net expenses of the national healthcare insurer were over 216 million euros for all products in these two classes. Costs calculated for this study equal the sum of the reimbursement of the health insurer and possible co-payments, and thus reflect list prices. For biologicals dispensed in the retail setting, list prices equal net costs and are therefore a suitable instrument to assess price competition. Due to the application of several cost-saving measures after loss of exclusivities of biological products, substantial savings are generated for the national health insurer. However, for biologicals used in the retail setting, we can assume that net costs in Belgium are higher compared to other countries where these products are part of tenders (Autoriteit Consument and Markt, 2019; IQVIA, 2020; Moorkens et al., 2021). This suggests there might be additional room for further price reductions. Yet, Belgian market data indicate that no further non-mandatory price erosion occurs after the application of these mandatory price reductions. Notwithstanding the efficient Belgian mechanism to generate short-term savings in the off-patent biologicals market, competition generated by biosimilars may contribute to further price reductions and associated savings. Although, one should keep in mind that achieving the lowest possible price for off-patent biologicals should neither be the goal to safeguard the sustainability of the market.

However, a particular volume of lower-cost competing products is required to induce price competition (IQVIA, 2018). Because biosimilar market shares are limited and the market remains dominated by originator products, there is no incentive for further spontaneous price reductions at the moment. If prices of lower-cost alternatives do not decline, there is no reason for reference products to reduce their prices either. Moreover, the already significant mandatory price reductions for both biosimilar and reference products limit the space for further price competition. Without volume guarantees for biosimilar products, this system is most likely not sustainable in the future as biosimilar manufacturers might opt out of the Belgian market. The retraction of filgrastim biosimilar Zarzio from the Belgian market in 2019, and the absence of insulin (lispro and aspart) or teriparatide biosimilars are the first indications of a non-sustainable situation in Belgium.

#### Hospital Setting

Off-patent biological medicines used in the hospital setting compete through public procurement procedures by hospitals or hospital buying groups. As mentioned above, evolutions in daily costs calculated in this analysis reflect the evolution in list price or reimbursement base. However, varying pricing mechanisms between the hospital and retail setting are present. Because competition for hospital products mainly occurs through confidential discounts in tenders, daily costs calculated for this study do not reflect net costs for the hospital. The observed lack of price competition on list price or reimbursement base is therefore expected. As known from data from several European countries, discounts as a result of competition in tenders can be substantial (Autoriteit Consument and Markt, 2019; IQVIA, 2020). It can be assumed that similar discounts are achieved in Belgian hospitals. Not surprisingly, interviewees frequently indicated that profits on pharmaceuticals are an important part of the hospital financing in Belgium. The latter was confirmed by the latest version of the yearly financial analysis of all Belgian hospitals, indicating that the purchasing of pharmaceuticals contributes to 20% of the hospital revenues in 2019. In terms of profit margins, it is the second most important source for Belgian hospitals (Belfius, 2020). A lowering of the list price (or reimbursement base) of hospital products might even be disadvantageous for the pharmaceutical company, because hospitals are often interested in selecting the product with the largest difference between reimbursement base and net price in tenders. This system encourages companies not to compete on list prices, but to save efforts for confidential discounts in tenders. Furthermore, Belgian list prices are the basis of reference prices in other European countries (Rémuzat et al., 2015; Kanavos et al., 2020). This might be an additional reason not to lower list prices. However, savings from the perspective of the Belgian health insurer are based on the lowering of list prices or reimbursement base. Therefore, several measures were put in place at the payer level to recover part of the hospital savings in tenders to the benefit of the national health insurer, such as lowering the reimbursement for biologicals for which biosimilar alternatives exist (National Institute for Health and Disability Insurance, 2019c). Any additional savings through price competition in tenders support the hospital financing. Both hospitals and the national health insurer are thereby benefiting from increased competition after biosimilar market entry.

### Shifts Toward New Versions of Existing Products, Second-Generation Products, or New Therapeutic Classes

For most therapeutic classes discussed in this article, a shift toward newer versions of existing products or second-generation products is observed. Moreover, in some cases, we observe a shift toward other therapeutic class products (i.e. JAK inhibitors). Clinical guidelines, developed by European scientific associations, generally do not recommend second-generation products or new therapeutic alternatives above existing off-patent biologicals, although we see them preferably being prescribed in Belgium (Jones et al., 2004; Curran and McCormack, 2012; Deicher and Hörl, 2012; Gossec et al., 2016; Cornes et al., 2018, 2020; Davies et al., 2018; Smolen et al., 2020; Torres et al., 2020). Most second-generation products or new therapeutic alternatives come at a higher cost, not always in proportion to the added value. Therefore, some clinical guidelines also highlight the responsibility of physicians to consider societal costs in their prescribing (Gossec et al., 2016; Davies et al., 2018; Smolen et al., 2020; Torres et al., 2020). Moreover, price reductions induced by biosimilar market entry alter the cost-effectiveness of biological therapy. Policymakers should therefore reconsider reimbursement modalities within the entire therapeutic class after the market entry of biosimilar medicines (Moorkens et al., 2020b; Simoens and Vulto, 2021). In order to stimulate the use of the best-value biological medicine, the Belgian reimbursement agency should bring their reimbursement modalities in line with the recommendation of European clinical guidelines to consider societal costs.

In Belgium, a specific instrument called “group revision” exists on the level of the Medicines Reimbursement Committee. This could be used to systematically reevaluate the reimbursement of all products within a therapeutic class after biosimilars have entered the market for certain products. For long-acting G-CSFs, this instrument has already proven to be suitable. The group revision of pegfilgrastim and lipegfilgrastim aligned their reimbursements in May 2020. However, as already mentioned, a new mandatory price reduction for pegfilgrastim caused again a price difference. At an even broader level, some off-patent biologicals compete with products of other therapeutic classes. Reevaluations based on group revisions do not impact other therapeutic classes. Hence, for such situations, a group revision would not be a suitable instrument. New approaches are needed in Belgium on how to reevaluate the reimbursement of new competing product classes, when the cost-effectiveness of competing products is altered after biosimilar market entry.

#### Retail Setting

In the retail setting, physicians seem to be less cost-sensitive as they tend to prefer newer alternatives such as the more concentrated insulin glargine formulation or JAK inhibitors. However, most recent clinical guidelines do not recommend preferential treatment with JAK inhibitors, nor with a specific TNF inhibitor in any of the approved indications (Gossec et al., 2016; Smolen et al., 2020; Torres et al., 2020). Also for the different insulin analog products, differences in hypoglycemia risk and glycemic efficacy are minimal despite the substantial cost differences between products (Davies et al., 2018). The reasons for this prescribing behavior remain unclear. The innovator climate in Belgium, along with marketing efforts of pharmaceutical companies, might have an influence. Especially when having in mind that both for off-patent anti-TNFs and insulin glargine, the same company markets the reference and the competing product (e.g. JAK inhibitor or more concentrated insulin glargine). These companies seem to have implemented impactful strategies to retain their part of the market.

Furthermore, different market dynamics between therapeutic domains were observed for TNF inhibitors. Shifts toward competing products are more pronounced in rheumatology and dermatology, compared with gastroenterology. For rheumatology, JAK inhibitors were marketed as a treatment alternative for existing TNF inhibitors and shifts to these products explain the decreasing volumes of SC anti-TNFs. IL inhibitors, on the other hand, have especially an important place in dermatology for the treatment of psoriasis. Presumably, treatment shifts toward IL inhibitors explain the decreasing use of existing TNF inhibitors in dermatology. In contrast to the limited added value of JAK inhibitors to treat rheumatoid arthritis, the arrival of IL-17/23 inhibitors (i.e. ustekinumab, secukinumab, ixekizumab) meant an improvement in the treatment of psoriatic diseases (Nast et al., 2017; Kerschbaumer et al., 2020; Smolen et al., 2020; Torres et al., 2020). Physicians often initiate new patients with IL inhibitors instead of TNF inhibitors to treat plaque psoriasis, despite their higher price compared to off-patent TNF inhibitors. Because of the higher costs of both JAK- and IL inhibitors compared to off-patent alternatives, such shifts largely offset the savings generated after biosimilar market entry. Physicians are responsible for treating patients in the best possible way with respect to increasing societal costs (Westhovens and Annemans, 2016; Sarzi-Puttini et al., 2019; Janke et al., 2020). Therefore, one could argue whether these treatments with limited added value should be used for all patients who meet the reimbursement criteria. Policymakers will have to look for ways how to stimulate cost-effective prescribing among Belgian physicians. For example, by increasing awareness among physicians about the societal costs of their prescription choices. Displaying these in comparison to treatment alternatives in the electronic prescribing system could be considered (Moorkens et al., 2020c). In addition, prescription budgets could also raise awareness and encourage cost-effective prescribing. In a country where healthcare is mainly publicly funded, physicians have the societal responsibility to consider the most cost-effective option or the best-value biological. Future research is required to better understand factors and interventions influencing the prescribing choices regarding off-patent biological medicines.

#### Hospital Setting

For biologicals dispensed in hospitals, other factors may influence the choice for new alternatives or second-generation products. Price is still one of the determining criteria when selecting a supplier in tenders, although other criteria generally play a role in Belgium (KMPG, 2019; Moorkens et al., 2020c). To ensure an equal level playing field between reference and biosimilar products in tenders, reimbursements have been aligned in past years. However, differences in reimbursement between products with distinct ATC-codes may be present in Belgium (e.g. long-acting G-CSFs). Regarding the subcutaneously administered versions of trastuzumab and rituximab, there may be several reasons for their preferred use in most hospitals. A first one is the hospitals’ preference for one single supplier for both the IV and SC formulation, because of logistic reasons. The tied selling of SC and IV products of the same manufacturer with additional discounts on the SC product, might also be important. Secondly, day hospital admissions contribute to the financing of Belgian hospitals (Belfius, 2020). The SC administration is associated with lower administration costs than the IV infusion, making them the preferred choice in some hospitals because they generate a larger hospital turnover (Franken et al., 2018; Altini et al., 2020). However, the savings in terms of drug acquisition costs after biosimilar market entry are believed to outweigh savings regarding administration costs (Barbier et al., 2019). A difference might exist between larger academic hospitals and smaller private hospitals, with different importance of day hospitalizations in their finances. The large influence of the Belgian hospital financing system on the off-patent biologicals market underlines the importance of the ongoing discussions about reforming hospital financing in Belgium (National Institute for Health and Disability Insurance (NIHDI), 2020b). In challenging times such as during the COVID-19 pandemic, Belgian hospitals are even more under pressure and their funding system is questioned more than ever.

### Strengths and Limitations of the Study

This study was the first to examine the Belgian off-patent biologicals market with quantitative market data, supplemented with qualitative insights from Belgian stakeholders. The combination of market data and stakeholder perceptions provides a comprehensive view of the Belgian market and explanations beyond the observations in the reimbursement data. This is the first study of its kind to provide a wider picture of the functioning of the market (both retail and hospital) for off-patent biologicals, including reference products, biosimilars, second-generation products, and other competing products from the same and other therapeutic classes. An analysis based on reimbursement data from the national health insurer (i.e. NIHDI) is the most appropriate way to gain an accurate insight into the Belgian market. Semi-structured interviews were preceded by exploratory stakeholder discussions with all relevant groups represented. This increased the relevance and depth of the chosen questions for the interview guide, resulting in more accurate insights. The relatively large sample of interviewees, given the limited number of Belgian stakeholders, increases the representativeness of the stakeholder insights captured in this study.

However, as already mentioned earlier, only reimbursed prices were available for this analysis. For hospital products that compete through tender procedures, it was therefore not possible to analyze the overall impact of competition in terms of cost savings because of the confidential nature of net prices in tenders. Qualitative research by means of semi-structured interviews has its limitations due to the nature of this method and the sample selection. Retrieved insights to explain the observations in the market are limited to the knowledge of the interviewed participants. The fact that interviewees were offered the opportunity to receive the interview guide in advance, could have increased the likelihood that answers were altered after consultation with third parties. The given insights could be influenced by what appears to be a societal acceptable answer from their perspective. The way in which this research could be intertwined with political decisions is also important in this regard.

### Future Perspectives

This study indicated a suboptimal Belgian market environment for off-patent biological and biosimilar medicines, thereby not fulfilling the potential benefits of best-value biologicals for a sustainable healthcare system. It also underlined that a *one-size-fits-all* approach for policymakers to stimulate a competitive market is not appropriate. Former research has shown differences in market dynamics between countries and even between regions (Ingrasciotta et al., 2015; Moorkens et al., 2019a; Moorkens et al., 2019b; Moorkens et al., 2020a). This study adds insights on variations in dynamics between therapeutic classes, indications, and healthcare settings (i.e. hospital or retail). Even differences between types of hospitals could be present, for example when looking at academic or private hospitals in Belgium.

Several biologicals that now contribute to a better treatment of different debilitating diseases will lose their market exclusivities in the coming years. This will mainly take place for targeted therapies in oncology and biologicals for the treatment of immune mediated inflammatory diseases (GaBi online, 2019b). Biologicals that will lose their exclusivities in the coming eight years, from 2019 onwards, had a total cost for the Belgian national health insurer of 656 million euros in 2018, without confidential rebates. Biosimilars are currently under development for most of these products and will provide the opportunity to reduce costs, thereby creating a more sustainable healthcare system in Belgium (Zorginstituut Nederland, 2020). For example, the Belgian health insurer recently reimbursed the first two bevacizumab biosimilars (BCFI, 2020a). The first IL-17/23 inhibitors (e.g. ustekinumab) will also lose their exclusivities, which might contribute to important future cost reductions and increased access to biological therapies for the treatment of psoriasis. Besides, new formulations of existing products are under development or will soon enter the market, such as the subcutaneous versions of infliximab and vedolizumab (GaBi online, 2019a; European Medicines Agency, 2020e). Competing product classes, like JAK inhibitors, will expand their indication profiles in the coming years, with newly approved indications and new products entering the market (Ferrante and Sabino, 2019; Westhovens, 2019). Notwithstanding the importance to stimulate the usage of best-value biologicals to support a sustainable healthcare system, policymakers should also pay attention to the incorrect or unnecessary usage of expensive biological therapies (Westhovens and Annemans, 2016; Ingrasciotta et al., 2021). Stimulating a competitive market is an important, yet not the only strategy to safeguard a sustainable healthcare system.

## Conclusion

To conclude, the off-patent biologicals market will continue to evolve in the coming years. Under current circumstances, the sustainability of the Belgian market is challenged by a combination of factors that were highlighted in this study. The Belgian hospital financing system has a major impact on this market and does not stimulate rational medicine usage. The business model of biosimilars and generics is based on larger volumes at lower prices. These lower prices are imposed by mandatory price reductions after their market entry, but there are currently not enough biosimilar volumes to perpetuate this model. It is the task of all Belgian stakeholders to create a climate where the benefits of best-value biologicals are captured, while ensuring a sustainable market that guarantees access for patients to biological therapies now and in the future.

## Data Availability

The original contributions presented in the study are included in the article/Supplementary Material, further inquiries can be directed to the corresponding author.
