# Supplementary material for: Off-Patent Biological and Biosimilar Medicines in Belgium: A Market Landscape Analysis
Source: Front Pharmacol. 2021 Apr 19;12:644187. doi: 10.3389/fphar.2021.644187 (PMC8091126; doi:10.3389/fphar.2021.644187)
Supplement: Supplementary file 1 [file datasheet1.docx]

Supplementary Material

# Interview guide

Breakdown of questionnaires per stakeholder group:

1. Physicians (and physicians’ organizations)
2. Patient organizations
3. Pharmacists
4. Nurses
5. Policy makers, regulators, health insurance funds, industry organizations, hospital managers

**Physicians (organizations)**

**General**

1. To what extent do you have experience with biosimilar medicines? (starting, switching)

Following questions may be adjusted according to the answer to this question.

**Information/education**

1. To what extent do physicians and patients have trust in biosimilar medicines in your environment?
   1. If there is a lack of trust. How could this be solved in the future?
   2. If not yet mentioned. Do you think it there is still need for extra initiatives about the provision of information/education regarding biosimilars towards physicians or patients?
   3. If yes, what kind of information would be needed?
   4. From who should this information best come?
2. To what extent are the current recommendations on switching sufficient? (FAMHP recommendation, clinical guidelines)
   1. What is still needed concerning recommendations or scientific advice regarding switching?
      1. From who should this type of information come?
3. When transitioning a patient to a biosimilar:
   1. What step-by-step plan is taken in your area when this happens? (if there is one)
   2. What information/communication is given to patients when switching from an original to a biosimilar medicine?
   3. Who is involved in this an what is everyone’s role?
   4. Which people should be involved more in the future?

**Prescription choice**

1. What are elements that determine your choice for a biosimilar or original biological medicine?
   1. What elements may determine your choice here for a certain product? What are reasons to switch or not to?
   2. If not yet covered, ask for the following:
      1. Product related: Effectiveness, safety, price, cost-effectiveness, manufacturer, administration, formulation
      2. Patient related: Indication, patient preferences, bio-naivety
      3. Other: Incentive, clinical guidelines, hospital formulas, preferences of key opinion leaders, amount of available information, added-services by companies
2. What kind of initiatives do you think are needed to further encourage biosimilars in the future?
3. What do you think about an incentive at the hospital level instead of at the level of the individual physician?
   - 1. How should this be organized for medicines dispensed in the ambulatory setting (adalimumab, etanercept)?
4. Since April 2019 the quota for cheap prescribing in Belgium also apply for medicines dispensed in hospitals for day-care patients.
   1. Is this measure sufficient? Why (not)?
   2. What can be done more in terms of prescription quota to create a more balanced market environment for biological medicines in hospitals?

**Ambulatory setting (public pharmacy)**

1. Are you aware of certain specific problems concerning biosimilars dispensed in public pharmacies? If yes, which one(s)?
   1. If not yet mentioned, ask for unavailability, contract with wholesalers, logistic problems, substitution for biological medicines
   2. What could be done to solve these issues?

**Price and reimbursement**

1. To what extent are the current price and reimbursement measures after biosimilar market entry leading to a competitive and sustainable market in Belgium? Why?
2. What do you think of the more recent introduced changes in reimbursement measures? (increased rice reduction, earlier price reduction regardless of biosimilar market entry).

**Closing**

1. As a final question, are there any other issues that you are thinking about that were not mentioned yet, but are important within the Belgian landscape regarding best-value biologicals? If yes, which ones?
   1. Are there other people to whom it might be interesting to talk regarding this study?

End with thanking the participant.

**Patient organizations**

**General**

1. What are your experiences as a patient or patient representative regarding biosimilars?

Following questions if applicable (depending on the answer to first question)

1. What are reasons for patients in your experience to choose for a biosimilar or originator biological?

**Information/education**

1. To what extent do patients have trust in your environment in biosimilar medicines?
   1. If there is a lack of trust. How could this be solved in the future?
   2. If not yet mentioned. Do you think it there is still need for extra initiatives about the provision of information/education regarding biosimilars towards patients?
   3. If yes, what kind of information would be needed?
   4. From who should this information best come?
2. Concerning switching/transitioning from an original biological to biosimilar:
   1. Which step-by-step plan was undertaken when switching?
   2. What kind of information/education was given to you concerning biosimilars?
   3. Who was involved in this process?
   4. Were you, in your opinion, sufficiently guided while switching/transitioning? Why (not)?

**Ambulatory setting (public pharmacy)**

1. Are you aware of certain specific problems concerning biosimilars dispensed in public pharmacies? If yes, which one(s)?
   1. If not yet mentioned, ask for unavailability, substitution.
   2. What could be done to solve these issues?

**Value-added services**

1. To what extent are you aware of certain added services that companies offer to patients regarding biological/biosimilar medicines? If yes, which ones?
2. If yes. What could be the impact of these services for patients?

**Closing**

1. As a final question, are there any other issues that you are thinking of that were not mentioned yet, but are important within the Belgian landscape regarding best-value biologicals? If yes, which ones?
2. Are there other people to whom it might be interesting to talk regarding this study?

End with thanking the participant.

**Pharmacists (organizations)**

**General**

1. To what extent do you have experience with (dispensing) biosimilar medicines?

Following questions will be adjusted according to the answer to this question.

**Information/education**

1. To what extent do pharmacists and patients have trust in biosimilar medicines in your environment?
   1. If there is a lack of trust. How could this be solved in the future?
   2. If not yet mentioned. Do you think it there is still need for extra initiatives about the provision of information/education regarding biosimilars towards pharmacists or patients?
   3. If yes, what kind of information would be needed?
   4. From who should this information best come?
2. To what extent are the current recommendations on switching sufficient? (FAMHP recommendation, clinical guidelines)
   1. What is still needed concerning recommendations or scientific advice regarding switching?
      1. From who should this type of information come?
3. When a patient switches from an original biological to a biosimilar:
   1. What step-by-step plan is undertaken in your environment? (if there is one)
   2. What information/communication is provided to the patient?
   3. Who is involved in the process of switching and who does what?
   4. Which people should be involved more in the future?

**Ambulatory setting (public pharmacy)**

*The three questions below only apply to retail pharmacists.*

1. Are pharmacists experiencing difficulties when dispensing biological or biosimilar medicines in public pharmacies? If yes, which?
   1. If not yet mentioned, ask for unavailability, contract with wholesalers, logistic problems, substitution for biological medicines
2. To what extent do you experience difficulties or practical issues when a patient is switching/transitioning from an originator biological to a biosimilar?
3. If any issues are present. What can be done more to solve these at the public pharmacy level?
   1. Who could play an important role here?

**Hospital financing: Procurement**

*Hospital financing questions only apply to hospital pharmacists*

1. What do you think about the current legal criteria that determine the choice for a particular tender?
   1. Are these criteria sufficiently clear and realistic?
   2. What other or additional criteria do you set for performing tenders from your perspective, with the purpose to creating a more sustainable and competitive market?
2. To what extent do certain value-added services play a role in the choice in tendering?
   1. What are examples of such services?
   2. To what extent is the principle of gainsharing already applied in your hospital? Does this create a more balanced and competitive market in your opinion?
   3. If present, how can gainsharing be optimized in your environment?
3. What do you think of multiple winning tenders? Is this desirable for a more competitive and sustainable Belgian market for biological medicines delivered in hospitals?
4. About the timing of tenders. For how long is a tender usually valid?
   1. Is this duration desirable for a competitive and sustainable market in your opinion?
   2. Is it practically feasible to reopen a tender when the first biosimilar enters the market? Why (not)?
      1. What would be a realistic period of time until a tender should be reopened after biosimilar market entry?
   3. What measures could be made to make a rapid reopening more feasible?
5. Currently, Belgian hospitals are increasingly tendering in groups of hospitals and no longer individually. What could be the impact of this on the biosimilar landscape?

**Hospital financing: General**

1. What are aspects in the current Belgian hospital financing system that may influence the uptake of biosimilars?
   1. What do you propose to change in the hospital financing system in order to limit the impact?
   2. What do you think of the recent changes in the hospital invoices (85% since 1 April 2019). How will this impact the market environment of biological medicines in hospitals?

**Price and reimbursement**

1. To what extent are the current price and reimbursement measures after biosimilar market entry leading to a competitive and sustainable market in Belgium? Why?
2. What do you think of the more recent introduced changes in reimbursement measures? (increased rice reduction, earlier price reduction regardless of biosimilar market entry).

**Closing**

1. As a final question, are there any other issues that you are thinking about that were not mentioned yet, but are important within the Belgian landscape regarding best-value biologicals? If yes, which ones?
   1. Are there other people to whom it might be interesting to talk regarding this study?

End with thanking the participant.

**Nurses**

**General**

1. To what extent do you, as a nurse, have experience with biosimilar medicines? (starting biosimilars, switching/transitioning)

Adjust following questions according to the answer on this question.

1. What is the role of a nurse when a patient starts with a biosimilar of switches to a biosimilar?
2. To what extent do you experience certain problems as a nurse when a patient is transitioning/switching to a biosimilar product?
   1. If present, what could be solutions for these problems?

**Information/education**

1. To what extent do nurses/patients/physicians have trust in biosimilar medicines in your experience?
   1. If distrust is present. How could this be solved in the future?
   2. If not yet mentioned. Do you think it there is still need for extra initiatives about the provision of information/education regarding biosimilars towards physicians, nurses or patients?
   3. Of yes, what kind of information would be needed?
   4. From who should this information best come?
2. When a patient switches from an original biological to a biosimilar:
   1. What step-by-step plan is undertaken in your environment? (if there is one)
   2. What information/communication is provided to the patient?
   3. Who is involved in the process of switching and who does what?
   4. Which people should be involved more in the future?

**Closing**

1. As a final question, are there any other issues that you are thinking about that were not mentioned yet, but are important within the Belgian landscape regarding best-value biologicals? If yes, which ones?
   1. Are there other people to whom it might be interesting to talk regarding this study?

End with thanking the participant.

**Industry organizations, policy makers, regulators, insurers, hospital managers, scientific associations**

*The following does not apply to hospital managers: information/education*

**Information/education**

1. To what extent do healthcare providers and patients have trust in biosimilar medicines in your environment?
   1. If there is a lack of trust. How could this be solved in the future?
   2. If not yet mentioned. Do you think it there is still need for extra initiatives about the provision of information/education regarding biosimilars towards healthcare providers or patients?
   3. If yes, what kind of information would be needed?
   4. From who should this information best come?
2. To what extent are the current recommendations on switching sufficient? (FAMHP recommendation, clinical guidelines)
   1. What is still needed concerning recommendations or scientific advice regarding switching?
      1. From who should this type of information come?

**Prescription choice**

1. What kind of initiatives do you think are needed to further encourage biosimilars in the future?
2. What do you think about an incentive at the hospital level instead of at the level of the individual physician?
   - 1. How should this be organized for medicines dispensed in the ambulatory setting (adalimumab, etanercept)?
3. Since April 2019 the quota for cheap prescribing in Belgium also apply for medicines dispensed in hospitals for day-care patients.
   1. Is this measure sufficient? Why (not)?
   2. What can be done more in terms of prescription quota to create a more balanced market environment for biological medicines in hospitals?

**Intellectual property**

1. To what extent do intellectual property or other exclusivities currently play a role in the market access of biological and biosimilar medicines?
   1. If there is an influence. What could be recommendations to create a more competitive environment?

**Ambulatory setting (public pharmacy)**

1. Are you aware of certain specific problems concerning biosimilars dispensed in public pharmacies? If yes, which one(s)?
   1. If not yet mentioned, ask for unavailability, contract with wholesalers, logistic problems, substitution for biological medicines
   2. What could be done to solve these issues?

**Hospital financing: Procurement**

*Hospital financing questions only apply to hospital pharmacists*

1. What do you think about the current legal criteria that determine the choice for a particular tender?
   1. Are these criteria sufficiently clear and realistic?
   2. What other or additional criteria do you set for performing tenders from your perspective, with the purpose to creating a more sustainable and competitive market?
2. To what extent do certain value-added services play a role in the choice in tendering?
   1. What are examples of such services?
   2. To what extent is the principle of gainsharing already applied in your hospital? Does this create a more balanced and competitive market in your opinion?
   3. If present, how can gainsharing be optimized in your environment?
3. What do you think of multiple winning tenders? Is this desirable for a more competitive and sustainable Belgian market for biological medicines delivered in hospitals?
4. About the timing of tenders. For how long is a tender usually valid?
   1. Is this duration desirable for a competitive and sustainable market in your opinion?
   2. Is it practically feasible to reopen a tender when the first biosimilar enters the market? Why (not)?
      1. What would be a realistic period of time until a tender should be reopened after biosimilar market entry?
   3. What measures could be made to make a rapid reopening more feasible?
5. Currently, Belgian hospitals are increasingly tendering in groups of hospitals and no longer individually. What could be the impact of this on the biosimilar landscape?

**Hospital financing: General**

1. What are aspects in the current Belgian hospital financing system that may influence the uptake of biosimilars?
   1. What do you propose to change in the hospital financing system in order to limit the impact?
   2. What do you think of the recent changes in the hospital invoices (85% since 1 April 2019). How will this impact the market environment of biological medicines in hospitals?

**Price and reimbursement**

1. To what extent are the current price and reimbursement measures after biosimilar market entry leading to a competitive and sustainable market in Belgium? Why?
2. What do you think of the more recent introduced changes in reimbursement measures? (increased rice reduction, earlier price reduction regardless of biosimilar market entry).

**Closing**

1. As a final question, are there any other issues that you are thinking about that were not mentioned yet, but are important within the Belgian landscape regarding best-value biologicals? If yes, which ones?
   1. Are there other people to whom it might be interesting to talk regarding this study?

End with thanking the participant.

# Supplementary Table 1. Overview of all discussed products in the quantitative analysis per therapeutic class

| **TNF-alpha inhibitors** |  |  |  |
| --- | --- | --- | --- |
| **Molecule name** | **Product name*** | **Reimbursed since** | **Dominant setting (hospital/retail)** |
| Infliximab | Remicade (RP) | 2001 | Hospital |
|  | Inflectra (B) | 2015 | Hospital |
|  | Remsima (B) | 2015 | Hospital |
|  | Flixabi (B) | 2017 | Hospital |
| Etanercept | Enbrel (RP) | 2002 | Retail |
|  | Benepali (B) | 2016 | Retail |
| Adalimumab | Humira (RP) | N/A | Retail |
|  | Amgevita (B) | 2018 | Retail |
|  | Hulio (B) | 2019 | Retail |
|  | Hyrimoz (B) | 2019 | Retail |
|  | Imraldi (B) | 2018 | Retail |
| Golimumab | Simponi | 2010 | Retail |
| Certolizumab pegol | Cymzia | 2010 | Retail |

| **JAK inhibitors** |  |  |  |
| --- | --- | --- | --- |
| Tofacitinib | Xeljanz | 2017 | Retail |
| Baricitinib | Olumiant | 2017 | Retail |

| **Insulin analogs (long-acting)** | | | |
| --- | --- | --- | --- |
| Insulin glargine | Lantus (RP) | 2004 | Retail |
|  | Toujeo | 2016 | Retail |
|  | Abasaglar (B) | 2016 | Retail |
| Insulin detemir | Levemir | 2005 | Retail |
| Insulin degludec | Tresiba | 2019 | Retail |

| **Granulocyte Colony-Stimulating Factors (G-CSF)** | | | |
| --- | --- | --- | --- |
| Filgrastim | Neupogen (RP) | 1992 | Hospital |
|  | Accofil (B) | 2010 | Hospital |
|  | Nivestim (B) | 2010 | Hospital |
|  | Tevagrastim (B) | 2010 | Hospital |
| Pegfilgrastim | Neulasta | 2005 | Hospital |
| Lipegfilgrastim | Lonquex | 2014 | Hospital |

| **Epoetins** |  |  |  |
| --- | --- | --- | --- |
| Epoetin alpha | Eprex (RP) | 1996 | Hospital |
|  | Binocrit (B) | 2008 | Hospital |
| Epoetin zeta | Retacrit (B) | 2009 | Hospital |
| Epoetin beta | NeoRecormon | 2000 | Hospital |
| MPG epoetin | Mircera | 2008 | Hospital |
| Darbepoetin | Aranesp | 2002 | Hospital |

| **Rituximab** |  |  |  |
| --- | --- | --- | --- |
| Rituximab IV | Mabthera (RP) | 2000 | Hospital |
|  | Truxima (B) | 2017 | Hospital |
| Rituximab SC | Mabthera | 2014 | Hospital |

| **Trastuzumab** |  |  |  |
| --- | --- | --- | --- |
| Trastuzumab IV | Herceptin (RP) | 2002 | Hospital |
|  | Herzuma (B) | 2018 | Hospital |
| Trastuzumab SC | Herceptin | 2014 | Hospital |

* The abbreviations RP and B are used to indicate whether it concerns a reference product (RP) or a biosimilar (B).

**Supplementary Table 2.** Overview of daily costs for all discussed products in Belgium in 2019. All costs are expressed as costs per DDD (€).

| **Molecule name** | **Product class** | **Daily costs (Cost per DDD in euros)** |
| --- | --- | --- |
| Infliximab reference | TNF inhibitor | 11,56067861 |
| Infliximab biosimilar(s) | TNF inhibitor | 11,053066 |
| Etanercept reference | TNF inhibitor | 24,00464016 |
| Etanercept biosimilar(s) | TNF inhibitor | 19,90561158 |
| Adalimumab reference | TNF inhibitor | 22,35869742 |
| Adalimumab biosimilar(s) | TNF inhibitor | 21,1081914 |
| Golimumab | TNF inhibitor | 33,47049892 |
| Certolizumab pegol | TNF inhibitor | 33,82637429 |
| Baricitinib | JAK inhibitor | 32,65858733 |
| Tofacitinib | JAK inhibitor | 31,06988056 |
| Insulin glargine reference | Long-acting insulin | 1,316972486 |
| Insulin glargine biosimilar | Long-acting insulin | 1,276572728 |
| Insulin glargine 300U | Long-acting insulin | 1,350519886 |
| Insulin detemir | Long-acting insulin | 1,684471726 |
| Insulin degludec | Long-acting insulin | 1,397802196 |
| Filgrastim reference | G-CSF | 50,39251454 |
| Filgrastim biosimilar(s) | G-CSF | 50,3413648 |
| Pegfilgrastim reference | G-CSF | 41,02855429 |
| Lipegfilgrastim | G-CSF | 53,18915512 |
| Epoetin alpha reference | Epoetin | 5,088778347 |
| Epoetin biosimilar(s) | Epoetin | 5,448412909 |
| Epoetin beta | Epoetin | 5,480463451 |
| MPG-epoetin | Epoetin | 5,853456185 |
| Darbepoetin alpha | Epoetin | 6,427409018 |
| Rituximab IV reference | Rituximab | 132,4275094 |
| Rituximab IV biosimilar(s) | Rituximab | 131,7210683 |
| Rituximab SC | Rituximab | N/A |
| Trastuzumab IV reference | Trastuzumab | 391,4018995 |
| Trastuzumab IV biosimilar(s) | Trastuzumab | 388,5699617 |
| Trastuzumab SC | Trastuzumab | N/A |
